# Quantum Efficiency Measurement and Modeling of Silicon Sensors Optimized for Soft X-ray Detection

**DOI:** 10.3390/s24030942

**Published:** 2024-01-31

**Authors:** Maria Carulla, Rebecca Barten, Filippo Baruffaldi, Anna Bergamaschi, Giacomo Borghi, Maurizio Boscardin, Martin Brückner, Tim A. Butcher, Matteo Centis Vignali, Roberto Dinapoli, Simon Ebner, Francesco Ficorella, Erik Fröjdh, Dominic Greiffenberg, Omar Hammad Ali, Shqipe Hasanaj, Julian Heymes, Viktoria Hinger, Thomas King, Pawel Kozlowski, Carlos Lopez Cuenca, Davide Mezza, Konstantinos Moustakas, Aldo Mozzanica, Giovanni Paternoster, Kirsty A. Paton, Sabina Ronchin, Christian Ruder, Bernd Schmitt, Patrick Sieberer, Dhanya Thattil, Konrad Vogelsang, Xiangyu Xie, Jiaguo Zhang

**Affiliations:** 1 Paul Scherrer Institut, Forschungsstrasse 111, 5232 Villigen, Switzerlandfilippo.baruffaldi@psi.ch (F.B.); anna.bergamaschi@psi.ch (A.B.); tim.butcher@psi.ch (T.A.B.); roberto.dinapoli@psi.ch (R.D.); erik.froejdh@psi.ch (E.F.); dominic.greiffenberg@psi.ch (D.G.); julian.heymes@psi.ch (J.H.); viktoria.hinger@psi.ch (V.H.); davide.mezza@psi.ch (D.M.); konstantinos.moustakas@psi.ch (K.M.); aldo.mozzanica@psi.ch (A.M.); kirsty.paton@psi.ch (K.A.P.); bernd.schmitt@psi.ch (B.S.); patrick.sieberer@psi.ch (P.S.); xiangyu.xie@psi.ch (X.X.); jiaguo.zhang@psi.ch (J.Z.); 2 Fondazione Bruno Kessler, Via Sommarive 18, 38126 Povo, Italy; giacomo.borghi@polimi.it (G.B.); boscardi@fbk.eu (M.B.); mcentisvignali@fbk.eu (M.C.V.); ficorella@fbk.eu (F.F.); ohammadali@fbk.eu (O.H.A.); paternoster@fbk.eu (G.P.); ronchin@fbk.eu (S.R.)

**Keywords:** soft X-ray sensors, entrance window, quantum efficiency

## Abstract

Hybrid pixel detectors have become indispensable at synchrotron and X-ray free-electron laser facilities thanks to their large dynamic range, high frame rate, low noise, and large area. However, at energies below 3 keV, the detector performance is often limited because of the poor quantum efficiency of the sensor and the difficulty in achieving single-photon resolution due to the low signal-to-noise ratio. In this paper, we address the quantum efficiency of silicon sensors by refining the design of the entrance window, mainly by passivating the silicon surface and optimizing the dopant profile of the n^+^ region. We present the measurement of the quantum efficiency in the soft X-ray energy range for silicon sensors with several process variations in the fabrication of planar sensors with thin entrance windows. The quantum efficiency for 250 eV photons is increased from almost 0.5% for a standard sensor to up to 62% as a consequence of these developments, comparable to the quantum efficiency of backside-illuminated scientific CMOS sensors. Finally, we discuss the influence of the various process parameters on quantum efficiency and present a strategy for further improvement.

## 1. Introduction

The Swiss Light Source (SLS) and Swiss X-ray free-electron laser (SwissFEL) at the Paul Scherrer Institut (PSI) have several beamlines working in the soft X-ray (SXR) energy range (200 eV–2 keV). The SXR energy regime covers the binding energies of the innermost electron shell (K-shell) of light elements (second and third rows of the periodic table), the second electron shell (L-shell) of 3d transition metals, and the third electron shell (M-shell) of rare earths. Thus, SXRs access valence-state electrons relevant to the structural, physical, and electronic properties of condensed matter. Tuning the X-ray energies near the K, L, or M absorption edges offers chemical and dichroic contrast. Techniques such as resonant soft X-ray scattering (R-SoXS) exploit the enhancement of scattering contrast and the high spatial resolution of SXR thanks to their short wavelengths (0.6 nm–6 nm) to reveal the nanostructure of thin films with light elements like organic semiconductors [1] or polymers [2]. Apart from R-SoXS, coherent lensless imaging techniques such as ptychography also benefit from the high spatial resolution and chemical contrast of SXR in the water window, between the carbon and oxygen absorption edges, to image biological samples with high contrast in its aqueous environment [3]. Furthermore, high-resolution imaging of magnetic nanostructures with SXR ptychography exploits the strong dichroic contrasts at the L-edges of 3d transition metals and M-edges of the lanthanides [4].

Charge-integrating and photon-counting hybrid detectors developed at the PSI are extensively used for hard X-rays. Specifically, charge-integrating hybrid detectors exhibit a linear response over a large dynamic range and fast frame rates adequate for the large instantaneous photon fluxes expected at SwissFEL [5] and at SLS2.0 after upgrading the SLS storage ring to a diffraction-limited light source [6]. However, the poor quantum efficiency (QE) of standard silicon sensors and the low signal-to-noise ratio (SNR) obtained at low photon energies (e.g., QE is <0.5% for 250 eV photons [7] and SNR is ∼5 for 800 eV photons with JUNGFRAU1.1 readout ASIC [8]) limit their suitability to use in the SXR energy range. Therefore, monolithic detectors are mostly used at SXR beamlines, specifically the backside-illuminated charge-coupled device (CCD), since the pixel size can be less than 20 µm and the noise can be as low as a few electrons. However, backside-illuminated CCDs show different shortcomings, namely, a slow frame rate (<10 frames per second), radiation damage, the requirement for an electronic shutter, limited area, and a decrease in QE for X-ray energies larger than 2 keV due to the thin silicon thickness (10 µm). Several detector systems based on CCD technology are being developed to increase the frame rate and improve the efficiency in the SXR regime. These include pnCCDs developed at the Max-Planck-Institut für extraterrestrische Physik and at PNSensor GmBH, which feature a 200 Hz frame rate for 256 × 512 pixels [9,10,11] and 83% QE for 250 eV photons [12]. As an alternative, back-illuminated complementary metal oxide semiconductor (CMOS) imagers have been optimized for SXR detection. Recent developments include a backside-illuminated scientific CMOS sensor commercialized by Gpixel, which achieves 48 frames per second at high gain and 62% QE [13], and the PERCIVAL detector, a monolithic active pixel sensor under development by a consortium of synchrotrons with a maximum frame rate of 300 Hz [14]. However, none of these systems can provide the same performance as hybrid detectors in the hard X-ray energy range, particularly the large detection area obtained by tiling several modules, a large dynamic range, radiation hardness, and high frame rates.

With the aim of developing a hybrid detector system with high quantum efficiency and single-photon resolution in the SXR energy range, the photon science detector group at the PSI started a collaboration with Fondazione Bruno Kessler (FBK) to develop silicon sensors with optimized performance [7]. In this paper, we report the investigation of the QE and internal quantum efficiency (IQE) of the produced sensors, analyzing the impact of different process parameters. The present paper is divided into five sections. After this introduction, the status of current sensors used for hard X-rays is presented in Section 2. The process technology used for producing the sensors, the experimental setup, and the quantum efficiency model used in this work are discussed in Section 3. Section 4 presents the results, and the findings are discussed in Section 5.

## 2. Hybrid Detectors for X-ray Detection

An outstanding advantage of hybrid detectors is that the material and process of the sensor and the readout electronics are optimized separately for specific applications. The readout electronics of the charge-integrating detectors developed at the PSI are fabricated in UMC110 CMOS technology and are optimized to achieve low noise, high frame rates, and a large dynamic range thanks to dynamic gain switching [15].

### 2.1. Sensors for Hybrid Detectors

**Figure 1:**
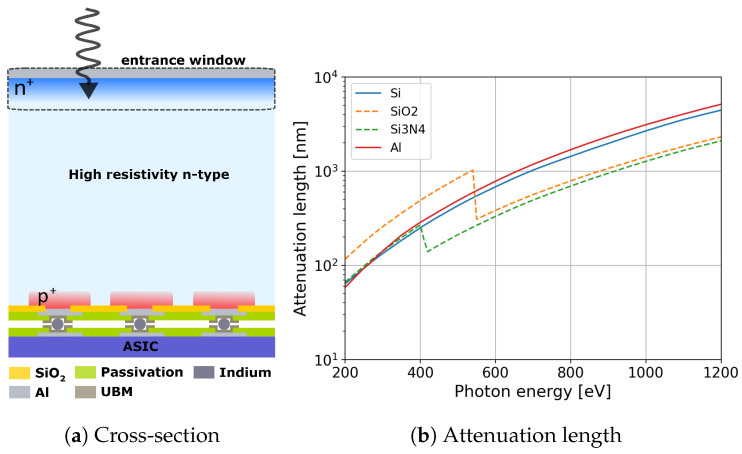
(**a**) Cross-section of a planar silicon sensor. (**b**) X-ray attenuation lengths in silicon, SiO_2_, Si_3_N_4_, and aluminum obtained from NIST database [18] for photon energies between 200 eV and 1250 eV.

The sensor consists of a semiconductor material in which X-rays are directly converted to electron–hole pairs through the photoelectric effect. In comparison to sensors with indirect conversion, which use phosphor screens as scintillators and detect the visible photons, direct-detection sensors offer a better spatial resolution and a larger signal-to-noise ratio (SNR) due to their higher conversion efficiency [16]. Conventional planar p^+^-n-n^+^ silicon sensors, schematically depicted in Figure 1a, are used to detect hard X-rays in the 5–20 keV energy range. The p^+^-n junction is located on the pixel side. The entrance window (EW), i.e., the sensor backplane where photons impinge on the detector, acts as an ohmic contact to bias the detector. It usually consists of a 1–2 µm thick aluminum layer on bare silicon and a 1–2 µm thick n^+^ region, which is partially undepleted and exhibits a very low charge collection efficiency. For photon energies above 20 keV, even 1 mm thick silicon sensors are quasi-transparent. Thus, high-Z materials such as GaAs or CdZnTe are employed to increase the detection efficiency [17]. However, the detection efficiency of silicon sensors decreases for X-ray energies lower than 3 keV because the majority of photons are absorbed in the inefficient EW. Thus, the QE of silicon sensors has to be improved in order to detect SXRs.

### 2.2. Quantum Efficiency

The QE of a silicon sensor for X-ray detection is defined as the probability of detecting an incident photon. An SXR photon primarily interacts with silicon through the photoelectric effect, generating charge carriers. The generated charge carriers drift towards the electrodes thanks to an externally applied bias voltage, inducing an electrical signal treated by the readout electronics. Some of the charge carriers can recombine in the silicon, which results in either a partial or complete loss of signal. The fraction of charge carriers that are finally collected by the readout electrode is defined as the charge collection efficiency (CCE) of the sensor, which is a function of the absorption depth of the photon.

For SXRs, the losses in QE are mainly caused by the complete loss of X-ray photons when they are absorbed in the inactive layers (ILs) on top of the silicon and the partial or complete loss of the generated charge carriers due to their recombination inside the silicon or at the Si/IL interface. Since the attenuation length of SXRs in silicon and aluminum is short (e.g., the attenuation length of 250 eV SXRs is 93 nm in silicon and aluminum, as shown in Figure 1b), the thickness of the inactive layers should be minimized. In addition, special care must be taken in the design of the highly doped n^+^ region to ensure the full collection of the generated charge carriers in this region. The lifetime of minority carriers (holes) in this heavily doped n^+^ is short since carriers are lost through Auger recombination, where the recombination rate is proportional to the carrier density [19]. Thus, the holes generated in this undepleted region, with a low electric field, cannot diffuse out of this region before recombination. In addition, the minority carriers can be trapped at the silicon/IL interface (silicon/aluminum for conventional sensors) since the surface recombination velocity at the silicon/aluminum interface is as high as $10^{7}$ cm/s, reducing the surface and the effective lifetimes of minority carriers [20]. Hence, the QE of standard sensors is less than 0.5% for 250 eV X-ray photons [7] due to these three loss mechanisms: (1) photon absorption in the aluminum layer, (2) surface recombination of the charge carriers at the Si/IL interface, and (3) Auger recombination of the charge carriers in the heavily doped n^+^.

In summary, three modifications have to be considered in order to increase the QE for SXRs:

- The IL needs to be thin and made of low-Z materials to reduce the probability of complete photon loss in the IL. The metallization thickness should be minimized in applications that require the use of this layer to avoid the detection of stray light.
- The quality of the silicon surface has to be improved by means of passivating the surface with a thermally grown oxide in order to reduce recombination near the silicon surface rather than using aluminum on bare silicon.
- The lifetime of minority carriers needs to be increased by reducing the doping concentration and the depth of the n^+^ region.

### 2.3. Signal-to-Noise Ratio

In addition to the optimization of the EW, further developments to increase the SNR and achieve single-photon resolution are necessary. For example, 250 eV X-rays only generate 70 electron–hole pairs, which is not enough to reliably separate the signal from the noise level (35 $e^{-}$ ENC r.m.s for JUNGFRAU v.1.1 in high gain [8]) and reject fake counts. In order to enhance the SNR, the signal amplitude can be increased by means of internal amplification within the silicon sensor [21].

In recent years, low-gain avalanche diode (LGAD) technology [22] has spread and has become a well-established technology that offers the ability to increase the signal by a factor of 10, without implementing any change in the readout electronics, at the cost of reducing the dynamic range of the detector.

In collaboration with FBK, we also produced an R&D sensor batch based on the inverse LGAD (iLGAD) technology [23] to improve the SNR. These iLGAD sensors also benefit from the optimization of the EW presented in this work, which is essential to obtain high QE for SXRs, as shown in detail in [24].

## 3. Materials and Methods

### 3.1. Optimization of the Process Technology

The sensors were fabricated on 6” n-type silicon wafers with an average resistivity of 7.5 kΩcm for wafers with the crystal orientation 〈100〉 and 13.5 kΩcm for wafers with the crystal orientation 〈111〉. The wafer thickness is 450 µm for the 〈100〉 wafers and 300 µm for the 〈111〉 wafers. The corresponding depletion voltages are 92 V for the 〈100〉 wafers and 23 V for the 〈111〉 wafers. Different parameters of the fabrication process of the sensors were varied to study their effects on the QE and the internal quantum efficiency (IQE). The different process variations are referred to as “splits”.

The p^+^ pixels were formed through boron implantation in the n-type silicon substrate. Thus, pn junctions were created on the pixel side. The backside of the sensor (EW side) consists of an n-type (n^+^) doped region, which was realized either through arsenic implantation or phosphorus diffusion. For both dopants, the backside was passivated with thermally grown SiO_2_, and a thin layer of Si_3_N_4_ was deposited on top.

**Figure 2:**
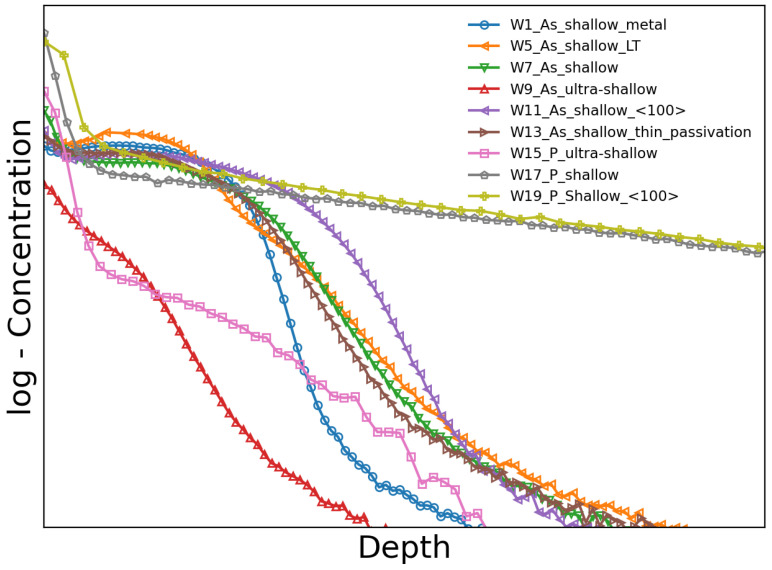
Dopant profiles obtained by secondary-ion mass spectrometry (SIMS) for the different splits of the sensor batch. The axis labels are not reported in order to protect the confidentiality of the process parameters.

A summary of the different process splits is presented in Table 1, and the dopant profile of each split is plotted in Figure 2. The dopant profiles were obtained by secondary-ion mass spectrometry at FBK. The ticks and labels on the axes are not reported due to the confidentiality of the dopant profiles. Figure 2 is supplied just to give a qualitative comparison between dopant profiles. Note that the dopant concentration is on a logarithmic scale.

Shallow and ultra-shallow dopant profiles of the n^+^ region were produced for both P and As dopants. The annealing temperature of the ultra-shallow dopant profile is low in comparison with the shallow dopant profile, except for W5, where the shallow arsenic implant was also annealed at a low temperature. The shallow dopant profile for both impurities was produced on wafers with both crystal orientations, i.e., 〈111〉 and 〈100〉. In contrast, the ultra-shallow dopant profile was only produced on wafers with a 〈111〉 crystal orientation. In the legends in the figures, the crystal orientation is 〈111〉 unless 〈100〉 is specified. The total thickness of the dielectric layers (SiO_2_ and Si_3_N_4_) is a few tens of nanometers. In order to explore the feasibility of reducing the dielectric thickness, it was thinned down in W13. In comparison to the splits with dielectric layers, no dielectric layer was placed on W1 in order to understand the impact of surface recombination. Instead, an aluminum layer with a thickness of a few hundred nanometers was deposited on top of the bare silicon to create an interface similar to conventional sensors.

Each split was electrically characterized after production. Table 2 presents the average volume leakage current densities of the test diodes placed at the periphery of the produced wafers. The splits produced on Si 〈100〉 show a larger volume leakage current density in comparison with splits on Si 〈111〉. This is caused by the difference in the impurities present in the starting material rather than the crystal orientation. Furthermore, the splits with low annealing temperatures feature lower volume leakage current densities in comparison with splits with high annealing temperatures. In general, the volume leakage current density is larger than that obtained by standard sensors (e.g., 0.65 µA/cm^3^). This increase in the volume leakage current density of sensors with a thin EW is due to two reasons: differences between the starting material and the larger lifetime in the n^+^ region in comparison with standard sensors. Thus, holes generated at the surface diffuse out of the n^+^ electrode, increasing the total current. Apart from the volume leakage current density, the surface recombination velocity at the Si/SiO_2_ interface on the pixel side was studied using gate-controlled diodes (GCDs). The surface recombination velocities for all the splits on Si 〈111〉 are also presented in Table 2. The surface recombination velocity for splits on Si 〈100〉 could not be calculated since the surface leakage current is too low compared to the diode leakage current. The surface recombination velocities of splits on Si 〈100〉 are expected to be lower than those of splits on Si 〈111〉 wafers. Overall, the values of surface recombination are similar to the values obtained for standard sensors (e.g., 6.42 cm/s).

### 3.2. Experimental Setup

**Figure 3:**
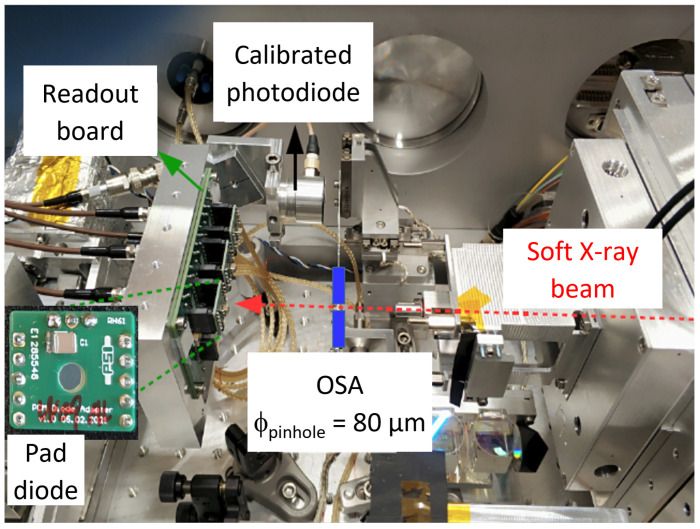
Experimental setup at the SIM beamline of the SLS. The inset shows one of the pad diodes under test mounted on a board with a 3 mm diameter hole on the irradiation side.

The measurement of the QE was performed at the Surfaces/Interfaces:Microscopy (SIM) beamline of the SLS. The SIM beamline can supply photons with energies ranging from 94 eV up to 2 keV, with photon fluxes $\leq 10^{13}$ photons/s/0.1%BW/400 mA and resolving power E/ΔE≤8000 [25]. The experimental setup is depicted in Figure 3. The SXR beam was provided by two 3.8 m Apple II undulators, followed by a plane-grating monochromator with a fixed-focus constant equal to 1.5 in order to reduce the high-order contamination. Afterwards, a microfocusing mirror focused the beam to the FLASH endstation. The vacuum chamber of the FLASH endstation was equipped with a detector stage, which was mounted on two precise motor stages right after an order-sorting aperture (OSA) stage, where an 80 µm pinhole was placed to collimate the beam, such that the beam was fully contained in the active area of the diode.

### 3.3. Quantum Efficiency Measurement

The quantum efficiency was measured using single pad diodes (presented in the inset of Figure 3) for each process split presented in Table 1. A readout board capable of accommodating an array of up to six pad diodes, biasing them and measuring their currents independently, was designed. The readout board was screwed onto an aluminum frame and tightened to the detector stage. An additional photodiode, specifically a passivated implanted planar silicon detector from Mirion technologies (PIPS PD-50-12-100-AM), was mounted on one side of the aluminum frame. The photodiode was calibrated by the Physikalisch-Technische Bundesanstalt (PTB) radiometry laboratory at the BESSY II synchrotron. The pad diodes under test were reverse-biased at 200 V. The current of the pad diodes and the calibrated photodiode were measured in dark conditions and under illumination with photon energies between 200 eV and 1250 eV. The photocurrent ($I_{ph}^{DUT}$) of each diode was obtained by subtracting the current without X-ray illumination ($I_{dark}^{DUT}$) from the current under illumination ($I_{X-ray}^{DUT}$). In order to reduce photocurrent variations caused by thermal drifts, the dark measurement was performed repeatedly at each photon energy just before the shutter was opened. The photocurrent of the pad diodes was normalized by the photocurrent of the calibrated photodiode ($I_{ph}^{cal}$). During the measurement at each energy, the variation in the photon flux was below 0.8%. Finally, the QE was calculated as a function of the photon energy, scaling the QE of the calibrated photodiode ($QE^{cal}$) to the normalized photocurrent

(1) $$QE^{DUT}\left(E\right)=QE^{cal}\left(E\right)\frac{I_{ph}^{DUT}\left(E\right)}{I_{ph}^{cal}\left(E\right)}=QE^{cal}\left(E\right)\frac{I_{X-ray}^{DUT}\left(E\right)-I_{dark}^{DUT}\left(E\right)}{I_{X-ray}^{cal}\left(E\right)-I_{dark}^{cal}\left(E\right)}$$

since the illuminated area was the same.

### 3.4. Modeling of the Quantum Efficiency

Considering SXRs with perpendicular incidence, the QE can be expressed as the probability of X-ray photons passing through the IL times the internal quantum efficiency (IQE), which can be obtained by integrating the product of the CCE and the probability of photons being absorbed at a given depth, x, over the entire sensor thickness:

(2) $$\begin{array}{ccc} QE(E) & = & e^{-t_{IL}/\lambda_{IL}\left(E\right)}\;IQE\left(E\right) \end{array}$$

(3) $$\begin{array}{ccc} IQE(E) & = & \int_{0}^{T}\frac{CCE\left(x\right)\cdot e^{-x/\lambda_{\mathrm{Si}}\left(E\right)}}{\lambda_{\mathrm{Si}}\left(E\right)}dx \end{array}$$

where *T* is the sensor thickness, $t_{IL}$ is the thickness of the inactive layer (e.g., of the aluminum or dielectric layer), and λ represents the attenuation lengths of X-rays either in silicon ($\lambda_{\mathrm{Si}}$) or in the IL on top of silicon ($\lambda_{\mathrm{IL}}$).

**Figure 4:**
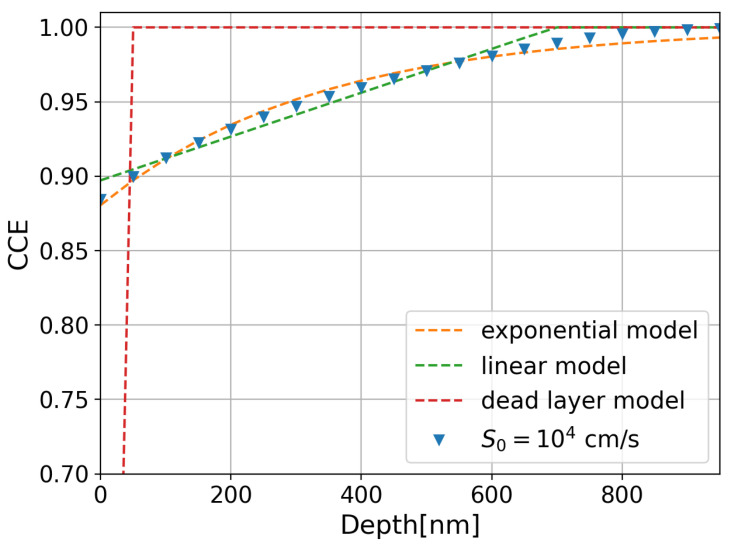
Calculation of the charge collection efficiency as a function of the depth after [27] for a surface recombination velocity of $S_{0}=1$×$10^{4}$ cm/s. The fits of the dead layer, linear, and exponential models are also plotted with red, green, and orange dashed lines.

Modeling the CCE as a function of the depth of absorption allows us to obtain an empirical expression that describes the measured QE and the IQE and quantitatively compare the process parameters of various sensors under test. In the present work, we describe the CCE as a function of two parameters:

(4) $$CCE\left(x\right)=1-\left(1-p_{0}\right)\cdot e^{-x/\tau }$$

where $p_{0}$ is the fraction of collected charge when X-ray photons are absorbed at the silicon surface (x=0), and τ is a decay constant that depends on the surface recombination velocity and the recombination rate in the n^+^ region. The dead layer model [13] and the linear model [26] are empirical CCE models commonly used for backside-illuminated CCDs, which exhibit low Auger and surface recombination. Unlike the dead layer and linear models, the exponential model used in this work (Equation (4)) is able to describe the approximated solution of the CCE proposed by Cuevas et al. [27], which is obtained by solving the current density and continuity equations in heavily doped regions, assuming a quasi-transparent n^+^ region. In this assumption, the holes diffusing towards the space-charge region experience bulk recombination, and the holes diffusing towards the surface only exhibit surface recombination. The blue triangles in Figure 4 show the calculated CCE using an analytical approximation for a Gaussian n^+^ dopant profile with a peak concentration $N_{0}=10^{20}$ ${\mathrm{cm}}^{-3}$ at the surface, σ=200 nm, and a surface recombination velocity $S_{0}=1$ × $10^{4}$ cm/s. The fits of the calculated CCE to the three models (dead layer, linear, and exponential models) are also presented in Figure 4 as dashed lines in red, green, and orange, respectively. The best agreement is obtained using the exponential model.

By substituting Equation (4) into Equation (2) and considering an infinitely thick sensor, which is realistic in the case of SXRs ($T\gg \lambda_{\mathrm{Si}}$), we obtain the IQE as a function of the energy:

(5) $$IQE\left(E\right)=\int_{0}^{\infty }\frac{e^{-x/\lambda_{Si}\left(E\right)}\cdot CCE\left(x\right)}{\lambda_{Si}\left(E\right)}dx=\frac{\left[\lambda_{Si}\left(E\right)+p_{0}\tau \right]}{\left[\tau +\lambda_{Si}\left(E\right)\right]}$$

Finally, we obtain the expression for the QE as a function of the energy by multiplying Equation (5) by the transmittance in the IL on top of the silicon, namely, aluminum or Si_3_N_4_ on top of SiO_2_:

(6) $$\begin{array}{c} QE\left(E\right)=e^{-t_{\mathrm{Al}}/\lambda_{\mathrm{Al}}\left(E\right)}\frac{\left[\lambda_{\mathrm{Si}}\left(E\right)+p_{0}\tau \right]}{\left[\tau +\lambda_{\mathrm{Si}}\left(E\right)\right]} \end{array}$$

(7) $$\begin{array}{c} QE\left(E\right)=e^{-t_{{\mathrm{SiO}}_{2}}/\lambda_{{\mathrm{SiO}}_{2}}\left(E\right)}\cdot e^{-t_{{\mathrm{Si}}_{3}N_{4}}/\lambda_{{\mathrm{Si}}_{3}N_{4}}\left(E\right)}\frac{\left[\lambda_{\mathrm{Si}}\left(E\right)+p_{0}\tau \right]}{\left[\tau +\lambda_{\mathrm{Si}}\left(E\right)\right]} \end{array}$$

where $t_{\mathrm{Al}}$, $t_{{\mathrm{SiO}}_{2}}$, and $t_{{\mathrm{Si}}_{3}N_{4}}$ are the thicknesses of the aluminum, SiO_2_, and Si_3_N_4_ layers, respectively. $\lambda_{\mathrm{Al}}$, $\lambda_{{\mathrm{SiO}}_{2}}$, and $\lambda_{{\mathrm{Si}}_{3}N_{4}}$ are the respective attenuation coefficients, which were taken from the NIST database [18].

We fitted the measured QE data with the expression in Equation (6) or Equation (7), depending on the IL on top of the silicon surface. Once we obtained the IL thickness, $p_{0}$, and τ, they were used to calculate the IQE.

## 4. Results

**Figure 5:**
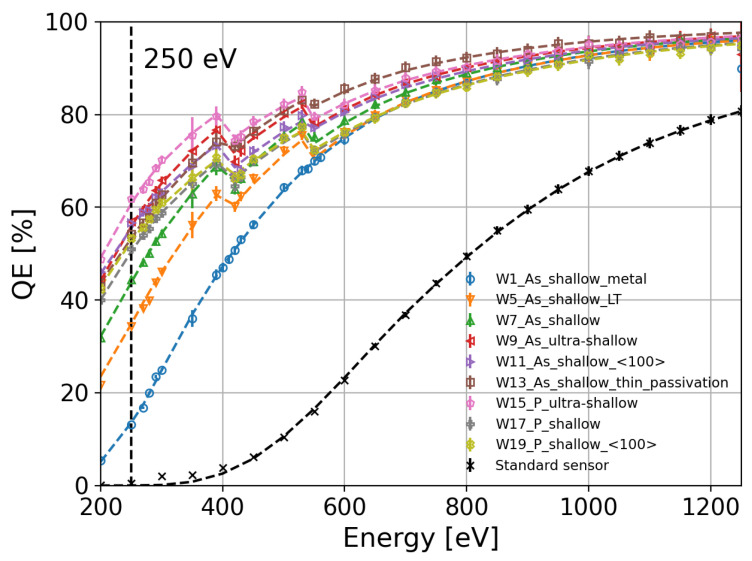
Quantum efficiency as a function of the photon energy for the splits described in Table 1 and a standard sensor used for hard X-rays (a).

Figure 5 summarizes the QE measurements for all the splits presented in Table 1. The diodes are compared to a standard sensor used for hard X-rays with an approximately 0.8 µm thick aluminum layer and an n^+^ implant that can extend to a few microns. The highest QE at 250 eV is above 62% for the W15 split featuring a phosphorus-diffused n^+^ region and an ultra-shallow dopant profile (pink markers). The improvement in the QE is evident compared with a standard sensor normally used for hard X-rays (black markers). Although the QE of a standard sensor can be improved by reducing the thickness of the aluminum layer and implanting a shallow n^+^ region using arsenic (W1, blue markers in Figure 5), the QE increases only up to 12% for 250 eV photons. In this case, the QE is dominated by the absorption of X-ray photons in the thin aluminum layer and the recombination of the charge carriers at the silicon/aluminum interface. In comparison to W1 with a silicon/aluminum interface, the splits that were passivated with dielectric layers (SiO_2_ and Si_3_N_4_) show a drop in QE in the absorption K-edges of nitrogen between 390 eV and 420 eV and oxygen between 530 eV and 550 eV. As an example, the QE of the W15 split with phosphorus diffusion and an ultra-shallow dopant profile reaches 80% at 390 eV and drops to 72% right above the binding energy of the nitrogen K-edge due to absorption in the Si_3_N_4_ layer. Similar behavior is observed around the oxygen K-edge due to the presence of a SiO_2_ layer.

Using the empirical model described by Equations (6) and (7), we fit the experimental QE data in order to extract the Al, SiO_2_, and Si_3_N_4_ thicknesses. The best fit for each split is plotted with dashed lines in Figure 5, and the values of the parameters obtained by the fit are listed in Table 3. Among the splits with shallow arsenic implantation, the one with a silicon/aluminum interface (W1, blue markers in Figure 5) and the split with low-temperature annealing (W5, orange markers in Figure 5) exhibit the lowest probability of charge collection at the surface. Note that the values of τ for splits with low p_0_ (W1, W5, W7, and W13) are small since the attenuation of the exponential term (1/τ) has to be faster in comparison with splits exhibiting larger charge collection probabilities at the surface (W9, W11, W15, W17, and W19).

**Figure 6:**
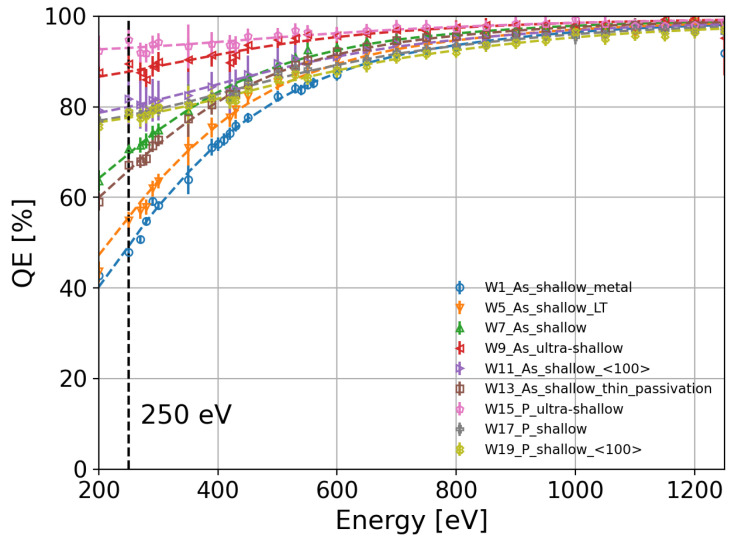
IQE as a function of the photon energy for the splits in Table 1.

**Figure 7:**
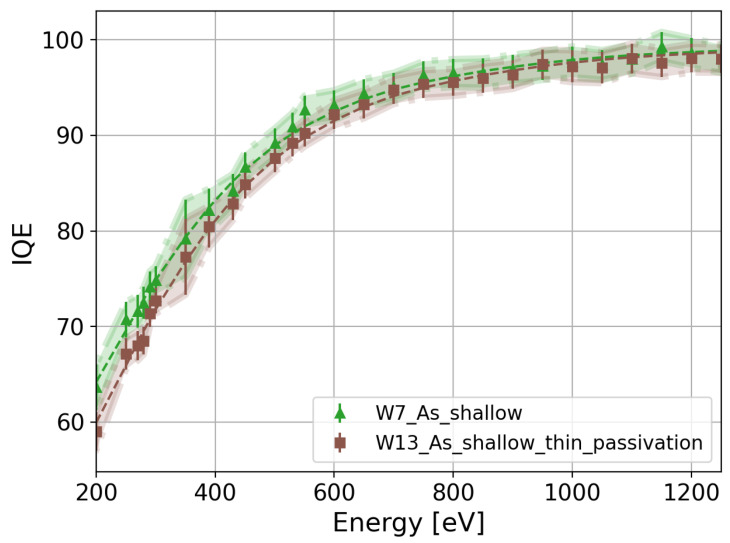
IQE comparison between W7 and W13, which have the same shallow As profile but differ in the dielectric thickness.

Once the fit parameters were determined, we calculated the IQE as a function of the photon energy for all the splits. A summary of the calculated IQE as a function of the photon energy for all the splits listed in Table 1 is presented in Figure 6. The IQE difference between W7 and W13, which differ only in the thickness of SiO_2_ and Si_3_N_4_, becomes negligible. The IQE differences lay in the error of the measurement for photon energies larger than 280 eV, as shown in Figure 7. Therefore, the estimation of the IL thicknesses and the calculation of the IQE using the QE model expressed in Equation (7) are consistent. The highest IQE is above 95% and 87% for the ultra-shallow phosphorus-diffused (W15, pink markers in Figure 6) and arsenic-implanted (W9, red markers in Figure 6) n^+^ regions, respectively. As observed in Figure 6, the IQE depends on the dopant profile, annealing temperature, crystal orientation, and doping process. In the following subsections, we will discuss the observed differences in the IQE as a function of the process parameters in detail.

### 4.1. Thermal Budget

**Figure 8:**
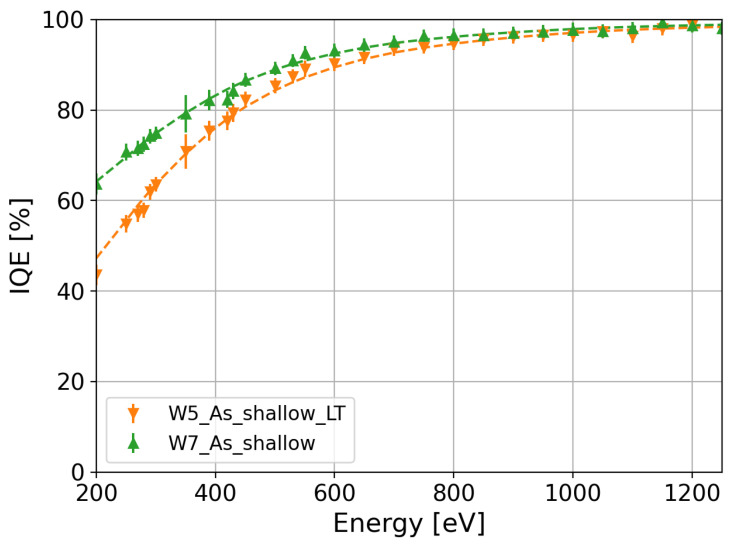
IQE comparison between low- and high-temperature annealing for the same As-implanted dopant profile.

During implantation, a distribution of point defects, vacancies, interstitials, and amorphous regions is generated in the silicon substrate. In addition to the silicon bulk damage, dangling bonds are also introduced at the SiO_2_/Si interface during implantation, which act as electron or hole traps. The damage induced by implantation depends on the species as well as the dose and energy of the implanted impurity. Post-implantation annealing moves the interstitial impurity atoms to a substitutional lattice site where the impurity is electrically active. Annealing also reduces crystal damage. High annealing temperatures are required for point-defect annihilation. However, shallow junctions require small thermal budgets. Thus, the area under the temperature–time curve has to be reduced by decreasing the annealing temperature and time. Since the thermal budget has an impact on the diffusion of the impurities and the annealing of the implantation damage, the shallow arsenic implantation was annealed at low (W5) and high (W7) temperatures to study its effect on the IQE. Note that at low temperatures, the annealing time was also reduced to further decrease the impurity diffusion. A comparison between W5 and W7 IQE values is shown with orange and green markers in Figure 8, respectively. The IQE of W5 with a low thermal budget is lower than that of W7 with a high thermal budget over the entire energy range of the measurement. In particular, the relative difference between W7 and W5 is greater than 25% at 250 eV. The decrease in the IQE at low temperatures can be explained by two factors. The first is the incomplete annealing of the implantation damage, which causes a loss of charge carriers through Shockley–Read–Hall (SRH) recombination at the silicon and SiO_2_/silicon interface. The second is the increase in the peak concentration of the dopant profile as a result of reduced diffusion in the W5 split, as shown in Figure 2 with orange markers. In comparison with W5, W7 (green markers in Figure 2) has a lower peak concentration. This increment in the peak concentration of the dopant profile produces a loss of charge carriers through Auger recombination, as discussed in Section 2.1. Thus, the temperature and time of annealing must be increased to reduce the loss of charge carriers in the n^+^ region and at the SiO_2_/silicon interface.

### 4.2. Dopant Profile

**Figure 9:**
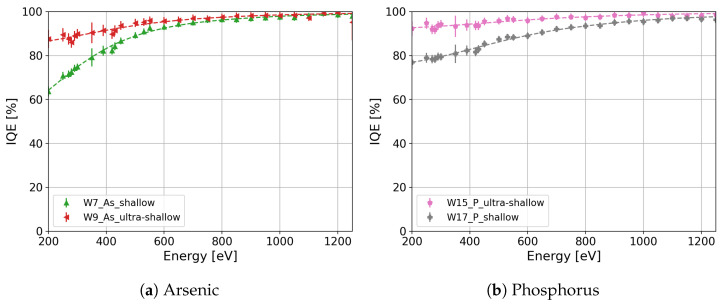
(**a**) IQE comparison between shallow and ultra-shallow dopant profiles for (**a**) the arsenic-implanted and (**b**) phosphorus-diffused n^+^ regions.

Another aspect influencing the loss of charge carriers in the n^+^ region is the dopant profile. Two different dopant profiles were produced, namely, shallow and ultra-shallow. The implantation dose and energy of arsenic were tuned to achieve shallow (W7, green markers in Figure 2) and ultra-shallow (W9, red markers in Figure 2) dopant profiles. In comparison, the shallow (W17, gray markers in Figure 2) and ultra-shallow (W15, pink markers in Figure 2) phosphorus profiles were adjusted through the use of different diffusion temperatures and times. The IQE of the shallow and ultra-shallow profiles of the arsenic-implanted split (W7 and W9 ) and of the phosphorus-diffused split (W17 and W15) are shown in Figure 9a and Figure 9b, respectively. As expected the IQE of the ultra-shallow profile is higher than that of the shallow profile, since the undepleted region in the ultra-shallow profile is thinner than in the shallow profile, and the peak concentration in the ultra-shallow dopant profile is lower than in the shallow dopant profile. Thus, the charge carriers generated in the ultra-shallow n^+^ have a higher probability of diffusing out of the highly doped region with a low electric field compared to those in the shallow profile. This prevents the charge carriers from recombining either at the silicon interface or in the undepleted region. The improvement in the IQE is above 20% at 250 eV for both ultra-shallow profiles compared with the shallow profiles.

### 4.3. Crystal Orientation

**Figure 10:**
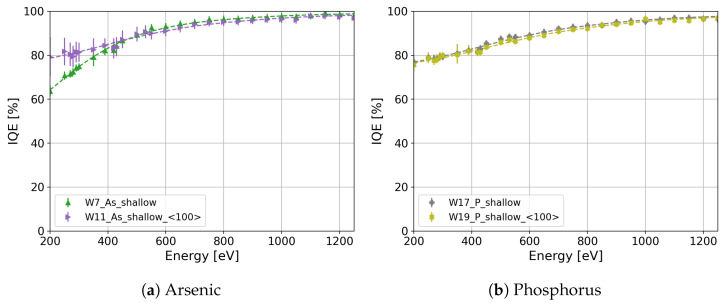
IQE comparison between Si〈111〉 and Si〈111〉 for (**a**) W7 and W11 with shallow arsenic implantation and (**b**) W17 and W19 with shallow phosphorus diffusion.

Under the same oxidation conditions, Si〈111〉 shows a higher density of interface states in comparison to Si〈100〉, according to [28,29,30], since the available bonds in Si〈111〉 are 2-fold larger than those in Si〈100〉. In this work, we study the influence of the crystal orientation on the IQE for sensors produced on 〈111〉 and 〈100〉 silicon substrates using the same process. Figure 10a presents a comparison between Si〈111〉 (W7, green markers) and Si〈111〉 (W11, violet markers) crystal orientations for the shallow arsenic n^+^ region. The IQE of W11 with a 〈100〉 crystal orientation is 1.14 times higher than that of W7 with a 〈111〉 crystal orientation for 250 eV photons. The observed enhancement in the IQE of the arsenic-implanted n^+^ region on Si〈100〉 (W11) is attributed to a higher density of interface states in Si〈111〉 compared with Si〈100〉 after implantation. The arsenic implantation causes a total ionization dose above 30 MGy in the dielectric layer, where a large difference in the interface states between Si〈111〉 and Si〈100〉 was observed and reported in [31]. It is worth mentioning that this improvement is not attributed to the arsenic profile differences since the concentration in W11 surpasses that of W7 throughout the entire depth range (Figure 2, W7 in green markers and W11 in violet markers).

**Figure 11:**
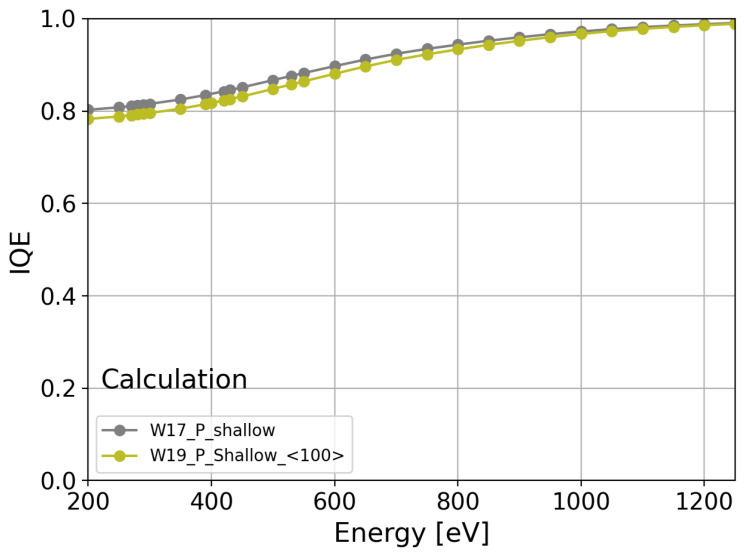
IQE calculation for the phosphorus-diffused profiles on Si〈111〉 (gray) and Si 〈100〉 (light green) using the quasi-transparent approximation [27], the phosphorus profiles, and surface recombination velocity equal to 1000 cm/s.

However, this difference is not observed in the phosphorus-diffused shallow n^+^ region, specifically for W17 (Figure 10b, gray markers) with a 〈111〉 crystal orientation and W19 (Figure 10b, light-green markers) with a 〈100〉 crystal orientation, indicating that the IQE is independent of the crystal orientation for the phosphorus-diffused process. However, the influence of the crystal orientation can be masked due to differences in the phosphorus profile (Figure 2, W17 in gray markers and W19 in light-green markers). To elucidate the effect of different phosphorus profiles on the IQE, the IQE was calculated by employing the quasi-transparent approximation of the CCE [27] and a surface recombination velocity of 1000 cm/s for both crystal orientations. Figure 11 presents the calculation of the IQE for W17 (gray markers) and W19 (light-green markers). As shown in Figure 11, the difference in the IQE due to the different doping profiles is less than 2.5% for 250 eV, which is less than the error in the IQE measurements of W17 and W19.

### 4.4. Implantation versus Diffusion

As mentioned in Section 4.1, trap levels in the silicon band gap are introduced during implantation, which causes the loss of charge carriers in the undepleted n^+^ region and at the SiO_2_/Si interface through SRH recombination when these are not annealed. In order to study the effect of implantation damage on the IQE, two doping processes were used to form the n^+^ region, namely, As implantation and P diffusion. Due to differences in the doping processes, the As and P profiles obtained for the same design of the n^+^ region are different. Therefore, IQE variations between As-implanted and phosphorus-diffused n^+^ regions are caused by implantation damage and differences in the dopant profile.

**Figure 12:**
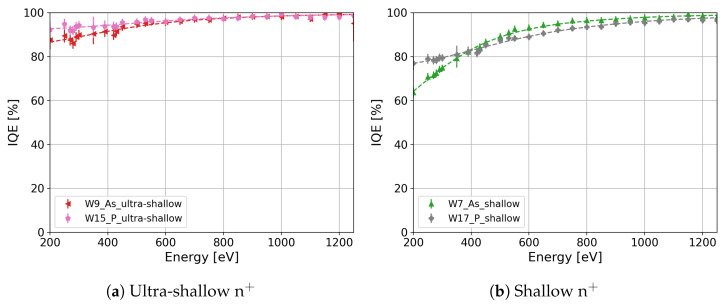
IQE comparison between impurity implantation and diffusion for (**a**) the ultra-shallow profiles and (**b**) the shallow profiles of the n^+^ region.

Two designs of n^+^ have been produced for both dopants: ultra-shallow and shallow designs. In the case of the ultra-shallow design of the n^+^ region, the As profile of W9 (Figure 2, red markers) and the P profile of W15 (Figure 2, pink markers) are slightly different. The profiles differ in the surface peak concentration and shape. The As concentration in W9 shows a lower peak concentration at the surface in comparison with the P concentration in W15. Furthermore, the As concentration in W9 decreases rapidly with the depth in comparison with the P concentration in W15. Although the P concentration in W15 is larger than the As concentration in W9 in the major part of the depth range, the IQE of W15 (Figure 12a, pink markers) is slightly larger than the IQE of W9 (Figure 12b, red markers) for photon energies lower than 600 eV. These minor variations in the IQE can be due to differences observed in the dopant profile. Similar behavior is observed in the As-implanted and phosphorus-diffused splits with the shallow designs of the n^+^ region, namely, in W7 (Figure 2, green markers) and W17 (Figure 2, gray markers), respectively. The As concentration at the surface of W7 is lower than the P concentration in W17. In addition, the As concentration in W7 decreases rapidly in comparison with the P concentration in W17. Although the As concentration in W7 decreases fast, there is a small range of depths where the As concentration is larger than the P concentration in W17. Figure 12b presents the IQEs of W7 and W17 in green and gray markers, respectively. The IQE of W7 for photon energies higher than 400 eV is moderately larger than the IQE of W17 due to the higher P concentration in W17 at deeper depths in comparison with the As concentration. However, the IQE of W7 for photon energies lower than 400 eV is smaller than the IQE of W17. In particular, the IQE of wafer 17 shows a 12% increase for 250 eV X-ray photons in comparison with the IQE of W7. The loss of charge carriers through Auger recombination can cause this decrease in the IQE of W7 since the As concentration in a small range of depths is higher than the P concentration. However, the observed difference between the IQEs of W7 and W17 for photon energies lower than 400 eV cannot be related just to the difference in the dopant concentration since the P concentration in W19 (Figure 2, light-green markers) is higher than the concentration in W7 for the entire range of depths, and the IQE of W19 is similar to that of W17 (Figure 9b). Thus, the decrease in the IQE of W7 for photon energies lower than 400 eV is also due to the incomplete annealing of the implantation damage, as mentioned in Section 4.1. This result stresses the importance of reducing the implantation damage by reducing the implantation dose and increasing the thermal budget.

## 5. Discussion

This paper presents experimental measurements of the QE and IQE of a dedicated sensor batch to optimize the entrance window in the energy range of SXRs (200 eV–1250 eV). In our study, we propose an empirical model to fit the measured QE and calculate the IQE for all the process splits. The IQE difference between two process splits with the same dopant profile but different thicknesses of SiO_2_ and Si_3_N_4_ is negligible, validating the estimation of the IL thicknesses using the proposed model.

**Figure 13:**
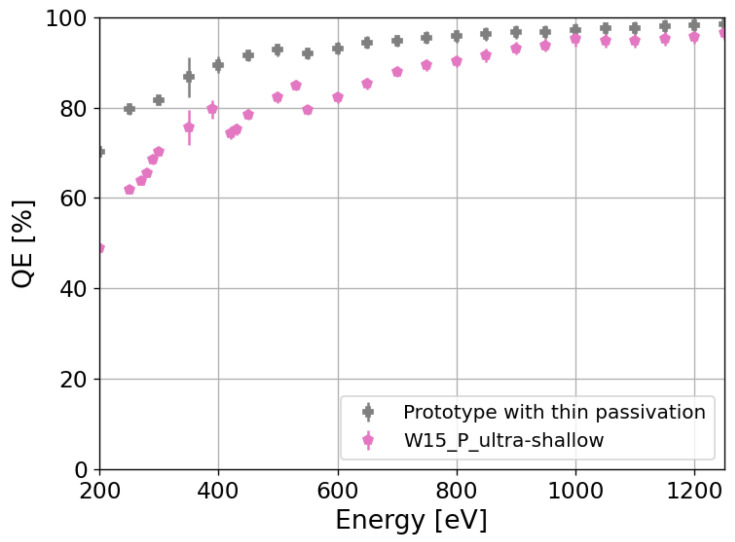
QE comparison between a prototype sensor with a ∼20 nm ${\mathrm{SiO}}_{2}$ layer and W15 from the FBK sensor batch under study.

We evaluate the influence of different dopant profiles, annealing temperatures, crystal orientations, and doping processes on the IQE as well. From the study of the IQE, we conclude that in order to reduce the losses in silicon, the improvement in the quality of the IL/Si interface by passivating the silicon surface with a thermally grown oxide is important. In addition, reductions in the peak concentration and depth of the n^+^ region, as well as the annealing of the implantation damage at high temperatures, further improve the IQE. In the specific case of an ultra-shallow design of the n^+^ region and a thermally grown oxide at the silicon surface, IQEs above 89% and 95% for 250 eV photons are obtained for the splits with arsenic implantation and phosphorus diffusion, respectively. Although the IQEs are close to 90%, the QEs are attenuated to 57% and 62% for 250 eV X-ray photons due to the additional absorption of X-ray photons in the SiO_2_ and Si_3_N_4_ layers. Hence, further improvement in the QE requires thinning the dielectric layers in order to reduce the absorption in these layers. This enhancement is exhibited in Figure 13, where the QE of the best split (W15) is compared to that of a prototype sensor with a ∼20 nm thick ${\mathrm{SiO}}_{2}$ layer. The QE of the prototype increases up to 80% for 250 eV X-rays. Further reduction in the total dielectric layer thickness (90% reduction) in future developments would increase the QE of sensors with ultra-shallow arsenic implantation and ultra-shallow phosphorus diffusion above 83% and 86% for 250 eV photons, respectively. Considering this reduction in the dielectric layers, the expected QE would be comparable to the QE of pnCCDs [12] and larger than backside-illuminated scientific CMOS sensors [13] (∼83% and ∼62% for 250 eV photons, respectively). Thus, the implementation of this future development together with iLGAD technology on silicon sensors would open the possibility of measuring single SXR photons with large-area hybrid detectors at high frame rates.

Hybrid pixel detectors implemented with this future development are foreseen to meet the stringent requirements for conducting time-resolved resonant inelastic X-ray scattering for 3d transition metals with soft X-rays at synchrotron and free-electron laser facilities, which requires a high quantum efficiency, good signal-to-noise ratio, fast frame rates, large detection area, and fine pixel pitch, as outlined in [32,33]. Additionally, these detectors offer the advantage of expediting data acquisition in various techniques, such as R-SoXS and SXR ptychography within the water window or near the L-edges of 3d transition metals at synchrotrons. Experiments will benefit from reduced acquisition time with the high-speed readout, higher spatial resolution and reduced radiation damage to the sample compared with scanning transmission X-ray microscopy (STXM), along with additional phase information with ptychography. Furthermore, these advancements in detector technology hold the potential for accelerating the inspection of lithography masks for ASIC foundries through ptychography using EUV photons, typically 92 eV [34], whose attenuation length in silicon aligns with that of 564 eV soft X-ray photons. Moreover, the high QEs of these sensors will improve the performance of hybrid detectors for low-energy electron detection, as in PEEM/LEEM applications, as discussed in [35]. The improved entrance window can allow acceleration voltages lower than 10 keV, leading to an improvement in the imaging resolution.

The increase in the QE of silicon sensors reported in this work is an important step to improve the performance of hybrid detectors for low-energy photon and electron detection and also exploit their versatility and reliability in this energy range.

## Figures and Tables

**Table 1:** Main specifications of different process variations for the EW R&D sensor batch characterized in this work.

Wafer	Si Orientation	n^+^ Dopant	Dopant Profile	Annealing Temperature	Surface Layer
W1	〈111〉	As	Shallow	High	Aluminum
W5	〈111〉	As	Shallow	Low	SiO_2_ and Si_3_N_4_
W7	〈111〉	As	Shallow	High	SiO_2_ and Si_3_N_4_
W9	〈111〉	As	Ultra-shallow	Low	SiO_2_ and Si_3_N_4_
W11	〈100〉	As	Shallow	High	SiO_2_ and Si_3_N_4_
W13	〈111〉	As	Shallow	High	Thin SiO_2_ and Si_3_N_4_
W15	〈111〉	P	Ultra-shallow	Low	SiO_2_ and Si_3_N_4_
W17	〈111〉	P	Shallow	High	SiO_2_ and Si_3_N_4_
W19	〈100〉	P	Shallow	High	SiO_2_ and Si_3_N_4_

**Table 2:** Volume leakage current density and surface recombination velocity on the pixel side of the sensor for the splits presented in Table 1.

Wafer	Volume Leakage Current Density (µA/cm^3^)	Surface Recombination Velocity (cm/s) (Pixel Side)
W1	5.1±0.5	1.799±0.017
W5	1.8±0.4	4.191±0.019
W7	4.4±0.3	4.281±0.014
W9	2.5±0.4	5.368±0.014
W11	9.1±0.6	-
W13	5.3±0.6	3.148±0.025
W15	3.2±0.5	5.659±0.016
W17	3.9±0.6	4.924±0.018
W19	7.3±0.5	-

**Table 3:** Parameters obtained from the fit of the QE data to Equation (6) for W1 and Equation (7) for the rest of the wafers.

Wafer	*t*_Al_ (nm)	*t*_SiO_2__ (nm)	*t*_Si_3_N_4__ (nm)	*p*_0_	τ (nm)
W1	121±22	-	-	0.020±0.009	100±9
W5	-	34±3	27±3	0.100±0.001	91±12
W7	-	33±2	27±2	0.41±0.03	100±8
W9	-	30±3	27±3	0.83±0.03	262±48
W11	-	17±3	25±3	0.73±0.03	368±33
W13	-	12±2	14±2	0.35±0.02	102±7
W15	-	31±2	24±2	0.92±0.02	589±157
W17	-	34±2	24±2	0.74±0.02	478±35
W19	-	30±2	21±2	0.74±0.02	585±45

## Data Availability

The data presented in this study are available on request from the corresponding author. The data are not publicly available due to the confidentiality of the process parameters.

## Abbreviations

The following abbreviations are used in this manuscript:

ASIC	Application-specific integrated circuits
CCD	Charge-coupled device
CCE	Charge collection efficiency
CMOS	Complementary metal oxide semiconductor
ENC	Equivalent noise charge
EW	Entrance window
FBK	Fondazione Bruno Kessler
GCD	Gate-controlled diode
IL	Inactive layer
iLGAD	Inverse low-gain avalanche diodes
IQE	Internal quantum efficiency
LGAD	Low-gain avalanche diodes
OSA	Order-sorting aperture
PSI	Paul Scherrer Institut
PTB	Physikalish-Technische Bundesanstalt
QE	Quantum efficiency
SHR	Shockley–Read–Hall
SIM	Surfaces/Interfaces:Microscopy
SLS	Swiss Light Source
SNR	Signal-to-noise ratio
SwissFEL	Swiss X-ray Free-Electron Laser
